# DataSUS: Uma Ferramenta Essencial para a Saúde Pública no Brasil

**DOI:** 10.36660/abc.20250123

**Published:** 2025-04-07

**Authors:** Marcia Koike

**Affiliations:** 1 Faculdade de Medicina da Universidade de São Paulo Departamento de Clínica Médica Laboratório de Investigação Médica da Disciplina de Emergências Clínicas São Paulo SP Brasil Laboratório de Investigação Médica da Disciplina de Emergências Clínicas, Departamento de Clínica Médica, Faculdade de Medicina da Universidade de São Paulo, São Paulo, SP – Brasil; 2 Instituto de Assistência Médica ao Servidor Público Estadual de São Paulo São Paulo SP Brasil Programa de Pós-Graduação em Ciências da Saúde do Instituto de Assistência Médica ao Servidor Público Estadual de São Paulo (IAMSPE), São Paulo, SP – Brasil

**Keywords:** Saúde Pública, Doenças Cardiovasculares, Sistema Único de Saúde

Desde sua criação em 1991, o Departamento de Informática do Sistema Único de Saúde (DATASUS) implementou sistemas de informação e suporte de informática necessários para o planejamento, operação e controle aos órgãos ligados aos SUS. Além disso, o DATASUS disponibiliza informações que podem auxiliar na análise da situação sanitária, na tomada de decisões baseadas em evidências e na elaboração de programas de saúde.^1^

A mensuração do estado de saúde da população começou com o registro de dados de mortalidade e sobrevivência, evoluindo para incluir informações epidemiológicas e de morbidade. Atualmente, dados sobre morbidade, incapacidade, acesso a serviços, qualidade da atenção, condições de vida e fatores ambientais são usados para criar Indicadores de Saúde, que são essenciais para quantificar e avaliar informações de saúde.^1^

Diante de um país enorme como o Brasil e com diferentes desafios regionais, a informatização dos sistemas de saúde foi gradual e representou um ganho para o processo de descentralização da saúde e para a avaliação epidemiológica dos agravos. Atualmente, o DATASUS inclui dados sobre mortalidade, internações, morbidades, assistência à saúde da população, cadastros das redes hospitalares e ambulatoriais, estabelecimentos de saúde, recursos financeiros e dados demográficos e socioeconômicos.^1^

Após mais de duas décadas de DATASUS, encontramos muitos artigos científicos que exploraram informações do banco de dados do DATASUS. Uma busca no site do Pubmed com a palavra DataSUS mostrou um pouco mais de 450 artigos relacionados, publicados entre 1998 e 2024.^2^ Observa-se uma evolução nas estratégias de avaliação dos dados e elaboração de artigos.

No início, muitos artigos descreveram perfis epidemiológicos das doenças, baseados no Sistema de Mortalidade (SIM)^3 – 6^ ou no Sistema de Internações (SIH).^7 , 8^ Com o decorrer do tempo, surgiram estudos mostrando as tendências temporais de uma ou duas décadas e projeções estatísticas, trazendo a maior compreensão da dinâmica de determinada doença e seus desfechos possíveis,^7 , 9 , 10^ bem como estudos de morbidade e os fatores de risco envolvidos.

Atualmente, o aprendizado de máquinas e a inteligência artificial trouxeram mais recursos e elegância aos achados oriundos do banco de dados disponibilizado pelo DataSUS, como por exemplo, um estudo de cohort publicado nesta edição.^11^

Neste estudo,^11^ autores exploraram os dados relacionados aos pacientes com artrite reumatoide em um estudo coorte, avaliando os tipos de Medicamentos Antirreumáticos Modificadores da Doença e a ocorrência de doenças cardiovasculares e os fatores associados aos desfechos cardiovasculares.

O acesso a um banco de dados nacional, com um grande contingente de pacientes, é um diferencial; além de se avaliar como os aspectos regionais podem afetar o desfecho do paciente. No entanto, reforço a importância do treinamento para operadores do sistema de informação em diferentes esferas, para o correto preenchimento da documentação ou na digitação destes, com critério e ética na elaboração dos documentos e transcrição. A acurácia das informações traz mais qualidade e uma valiosa oportunidade para estratégias de análise.^12^ Estudos de associações, projeções ou surgimento de emergências em saúde já estão em curso em outros países, como o Canada, que informatizou o sistema de saúde e se utiliza do banco de dados disponível para as estimativas.^13^
