# Supplementary material for: DataSUS: Uma Ferramenta Essencial para a Saúde Pública no Brasil
Source: Arq Bras Cardiol. 2025 Apr 7;122(2):e20250123. [Article in Portuguese] doi: 10.36660/abc.20250123 (PMC12040387; doi:10.36660/abc.20250123)
Supplement: Translation

# Translation: Datasus: An Essential Tool for Public Health in Brazil

**DOI:** 10.36660/abc.20250123i

**Authors:** Marcia Koike

**Affiliations:** 1 São Paulo SP Brasil Laboratório de Investigação Médica da Disciplina de Emergências Clínicas, Departamento de Clínica Médica, Faculdade de Medicina da Universidade de São Paulo, São Paulo, SP – Brasil; 2 São Paulo SP Brasil Programa de Pós-Graduação em Ciências da Saúde do Instituto de Assistência Médica ao Servidor Público Estadual de São Paulo (IAMSPE), São Paulo, SP – Brasil

**Keywords:** Public Health, Cardiovascular Diseases, Unified Health System

Since its creation in 1991, the Information Technology Department of the Unified Health System (DATASUS) has implemented information systems and IT support necessary for the planning, operation, and control of institutions linked to the SUS. Furthermore, DATASUS provides information that can assist in analyzing the health situation, making evidence-based decisions and developing health programs.^1^

Measuring the population's health status began with recording mortality and survival data evolving epidemiological and morbidity information. Currently, data on morbidity, disability, access to services, quality of care, living conditions, and environmental factors are used to create Health Indicators essential for quantifying and evaluating health information.^1^

Faced with a huge country like Brazil and with different regional challenges, the computerization of health systems was gradual and represented a gain for the process of health decentralization and epidemiological disease assessment. Currently, DATASUS includes data on mortality, hospitalizations, morbidities, population health care, records from hospital and outpatient networks, health establishments, financial resources, and demographic and socioeconomic data.^1^

After more than two decades of DATASUS's existence, we found many scientific articles that explored information from the DATASUS database. A search on the Pubmed website using DataSUS as a keyword showed just over 450 related articles published between 1998 and 2024.^2^An evolution can be observed in data evaluation and article preparation strategies.

Initially, many articles described epidemiological profiles of diseases based on the Mortality Information System (SIM)^3 – 6^or the Hospital Admission System (SIH).^7 , 8^Over time, studies emerged showing temporal trends over one or two decades and statistical projections, bringing a greater understanding of the dynamics of a given disease and its possible outcomes,^7 , 9 , 10^as well as studies of morbidity and the risk factors involved.

Currently, machine learning and artificial intelligence have brought more resources and elegance to the findings in the database made available by DataSUS as a cohort study published in this edition.^11^

In this study,^11^the authors explored data related to patients with rheumatoid arthritis in a retrospective cohort study, evaluating the types of disease-modifying antirheumatic drugs and the occurrence of cardiovascular diseases and factors associated with cardiovascular outcomes.^11^

Access to a national database with a large contingent of patients is a differentiator, in addition to evaluating how regional aspects can affect the patient's outcome. However, I reinforce the importance of training information system operators in different spheres to correctly fill out documentation or type it, with criteria and ethics in preparing documents and transcription. The accuracy of information brings more quality and a valuable opportunity for analysis strategies.^12^Studies of associations, projections, or emergence of health emergencies are already underway in other countries, such as Canada, which has computerized the health system and uses the available database for estimations.^13^
